# Surgically Resected Cardiac Angiosarcoma: Survival Analysis from the National Cancer Database

**DOI:** 10.3390/jcm12247764

**Published:** 2023-12-18

**Authors:** Mohamed Rahouma, Massimo Baudo, Sherif Khairallah, Christopher Lau, Mario Gaudino, Magdy M. El-Sayed Ahmed, Akshay Kumar, Roberto Lorusso, Stephanie L. Mick

**Affiliations:** 1 Cardiothoracic Surgery Departments, Weill Cornell Medicine, New York, NY 10065, USA; massimo.baudo@icloud.com (M.B.); smk4005@med.cornell.edu (S.K.); chl9077@med.cornell.edu (C.L.); mfg9004@med.cornell.edu (M.G.); slmick@med.cornell.edu (S.L.M.); 2 Surgical Oncology Department, National Cancer Institute, Cairo University, Cairo 12613, Egypt; 3 Cardiac Surgery Department, Spedali Civili di Brescia, University of Brescia, 25121 Brescia, Italy; 4 Cardiothoracic Surgery Department, Mayo Clinic, Jacksonville, FL 55905, USA; elgoharymagdy@yahoo.com; 5 Department of Surgery, Faculty of Medicine, Zagazig University, Zagazig 44519, Egypt; 6 Department of Cardiothoracic Surgery, Heart and Lung Transplantation, Mechanical Circulatory Support and ECMO, New York University Langone Health, New York, NY 10016, USA; drakshay82@gmail.com; 7 Department of Cardio-Thoracic Surgery, Maastricht University Medical Centre, Maastricht University, 6211 LK Maastricht, The Netherlands; roberto.lorussobs@gmail.com; 8 Cardiovascular Research Institute Maastricht, 6229 ER Maastricht, The Netherlands

**Keywords:** national cancer database, late mortality, malignant cardiac tumors, angiosarcoma, cardiac surgery

## Abstract

Angiosarcoma is a rare type of soft-tissue sarcoma arising from endothelial cells. It is considered ‘high-grade’ by definition, reflecting its aggressive behavior. We sought to investigate the role of surgery in cardiac angiosarcoma, identify late mortality predictors, and identify interactions with other modalities in its treatment using a national dataset. The 2004–2017 National Cancer Database was reviewed for patients with primary cardiac angiosarcoma. Late mortality predictors were evaluated with Kaplan–Meier curves and Cox regression analysis. Surgery in primary cardiac angiosarcoma was performed in 130 patients (median age 50.5 years; female sex 36.9%). The median follow up was 72.02 months, with a median overall survival (OS) of 14.32 months. In patients treated with surgery in combination with other modalities compared with those treated with surgery alone, median OSs were 17.28 and 2.88 months, respectively (log-rank = 0.018). Older patients (age > 57 years) experienced lower OS compared to those with an age < 57 (log-rank = 0.012). This may be partially explained by the difference in treatment strategies among age groups: those with increasing age, less surgery (*p* = 0.037), and less chemotherapy (*p* < 0.001) were chosen. With multivariable Cox regression analysis, age and race other than white or black were identified to be significant independent predictors of late mortality. Cardiac angiosarcoma has poor overall survival, and our findings should further encourage the use of surgery in combination with other therapeutic modalities in treating such an aggressive disease whenever possible.

## 1. Introduction

Primary cardiac malignancies are rare and far less common than secondary cardiac tumors [1,2]. Within primary cardiac tumors, approximately 10% exhibit a malignant trait [1]. Patients are typically asymptomatic initially, but can rapidly progress to decompensated heart failure and systemic symptoms of malignancy [3]. Indeed, dyspnea emerged as the predominant presenting symptom in cases of cardiac tumors [1]. Among primary malignant cardiac tumors, 95% are sarcomas of varying histologic types [4]. Primary cardiac sarcomas, which derive from endothelial cells, are generally located in the right atrium [5] and have an unfavorable prognosis [1] due to their aggressive biological behavior, which particularly impacts elderly patients [6]. Nevertheless, with the development of diagnostic and combination therapeutic approaches including surgery, chemotherapy, and radiotherapy, the survival of these patients has been improved [7]. At present, there is no definitive clinical evidence that establishes optimal management.

Angiosarcoma is the most common form of primary cardiac sarcoma, accounting for about one third of such cases [8]. This is to be contrasted to non-cardiac sarcomas, in which angiosarcoma makes up only a tiny fraction of these neoplasms [9]. Primary cardiac angiosarcomas are characterized by frequent infiltration into surrounding cardiac tissues and rapid progression to metastasis [10]. Metastatic spread is associated with a worse prognosis of cardiac angiosarcoma [11] and has been identified as a predictor of worse late outcomes compared to other sarcomas [12].

The aim of the present study was to investigate patients undergoing surgery for primary cardiac angiosarcoma, to identify predictors of late mortality, and to characterize the role of surgery in its treatment using the National Cancer Database (NCDB).

## 2. Materials and Methods

### 2.1. Study Population

The NCDB is an oncology database managed by the American Cancer Society and the American College of Surgeons. The NCDB was retrospectively reviewed for primary cardiac tumors from 2004 to 2017. All cases with more than one malignant neoplasm over the lifetime of the patient, those with missing survival time or status, non-angiosarcoma, and unknown treatment modality (surgery, radiotherapy, or chemotherapy) were excluded (Figure S1).

### 2.2. Outcomes of Interest

The following variables were evaluated: age, sex, race, insurance status, median income quartile, area (metropolitan/urban/rural counties), education (expressed as no high school graduate quartile, which gives a measure of the number of adults aged 25 or older in the patient’s zip code who did not graduate from high school and is categorized as equally proportioned quartiles among all US zip codes), great circle distance (defined as the distance, in miles, between the patient’s residence and the hospital that reported the case), Charlson/Deyo comorbidity condition (CDCC, categorized as 0 or 1 vs. 2 or 3), year of diagnosis, grade, tumor size, analytic stage, facility type (categorized as 1—community facilities (which included (A) academic/research program and (B) integrated network cancer program) and 2—academic/integrated facilities (which included (A) community cancer program and (B) comprehensive community cancer program)), different treatment patterns of surgery, radiotherapy, and chemotherapy, 30-days mortality, and late mortality.

The primary outcome was the assessment of late mortality differences among cardiac angiosarcoma patients who underwent surgery overall and by progressively longer survival times (short: <2, intermediate: 2–3, and late: ≥3 years). The secondary outcome was to investigate predictors of late mortality in this subset of patients, interactions with other modalities in its treatment, and age-related survival differences.

### 2.3. Statistical Analysis

Categorical data were summarized as percentages; significant differences or associations were analyzed using the X^2^ test or Fisher’s exact test, accordingly. Continuous variables were presented as mean ± standard deviation (SD) or median (interquartile range, IQR) depending on normality. Associations of quantitative data were analyzed with Student’s *t*-test and with the non-parametric Mann–Whitney U-test.

Long-term mortality was assessed using Kaplan–Meier curves and compared across groups using a log-rank test. Hazard ratios (HRs) were estimated using Cox regression analysis. Schoenfeld’s residuals were used to confirm the proportional hazards assumption. Independent predictors of overall late mortality were assessed using multivariable Cox regression. Median follow up time was assessed using the reversed Kaplan–Meier curves method. Maximally selected rank statistics, using “maxstat” and “survminer” R packages, were used to identify the best cutoff for age in relation to late mortality. This is an outcome-oriented method providing a value of cutoff that corresponds to the most significant relation with outcome.

A two-tailed value of *p* < 0.05 was taken to indicate statistical significance. Statistical analysis was performed using R version 4.1.1 within RStudio.

## 3. Results

During the considered study period, 110,991 malignant soft tissue tumors were identified, 907 of which were malignant cardiac tumors. One hundred and seven cases were excluded due to the presence of a sequence of more than one malignant neoplasm over the lifetime of the patient, and 64 patients were excluded due to missing survival time or status. Among the remaining 736 patients, a total of 322 angiosarcoma cases were identified. An additional 28 cases were removed as the treatment strategy was not specified (surgery, chemotherapy, or radiation). Finally, 164 patients who did not undergo surgery were excluded. Overall, a total of 130 patients were included (Figure S1).

The median age was 50.5 years (interquartile range (IQR): 37 to 62 years), and 36.9% (n = 48) were females (Table 1). The majority of patients in this cohort were white (69.3%), and private insurance was the most common insurance status (68.5%). The vast majority of the included patients (87.3%) resided in metropolitan areas, while only 4.0% were in rural areas. Proportions of different education categories were almost equal, but there was an increasing frequency from low to high median income status. Median great circle distance was 18.3 miles [IQR 7.7, 50.8]. The comorbidity index was low (0 or 1) in 91.5% of the cohort. Stage x/0/1 represented 56.2% of the patients, while stage II and stage III/IV represented 11.5% and 32.3%, respectively. Chemotherapy and radiotherapy were performed in 67.7% and 16.2% of patients, respectively.

No significant differences were reported between short, intermediate, and late survivors (<2, 2–3, and ≥3 years, respectively) (Table 1), nor when subdivided into short-term survivors (<3 years) and late-term survivors (≥3 years).

### Survival Analysis

The 30-day mortality was 10.8% in the overall population, which corresponded to 14.9% in the short survivor group.

**Figure 1:**
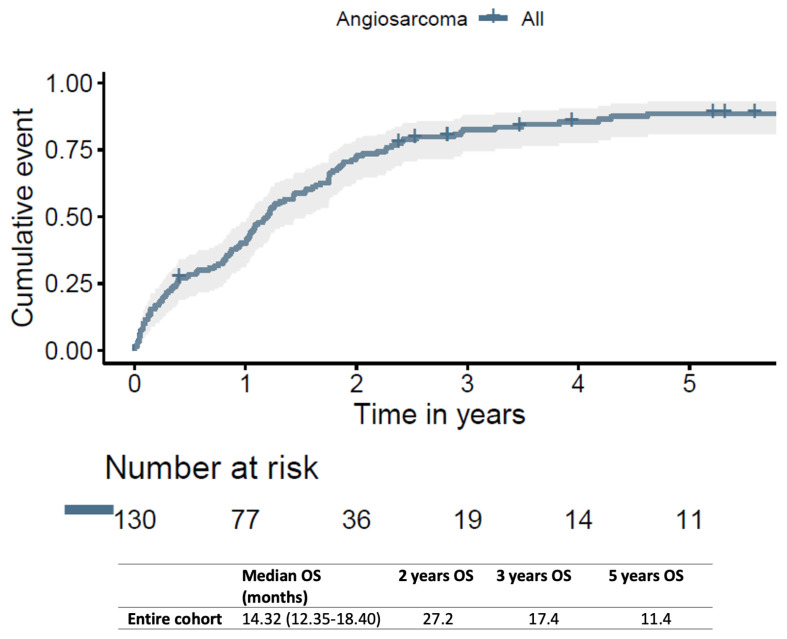
Cumulative late mortality among the entire cohort.

The median follow up was 72.02 months, with a median overall survival (OS) of 14.32 months (95% confidence interval [CI]: 12.35–18.40). The 2-, 3-, and 5-year OSs were 27.2, 17.4, and 11.4 months, respectively (Figure 1).

**Figure 2:**
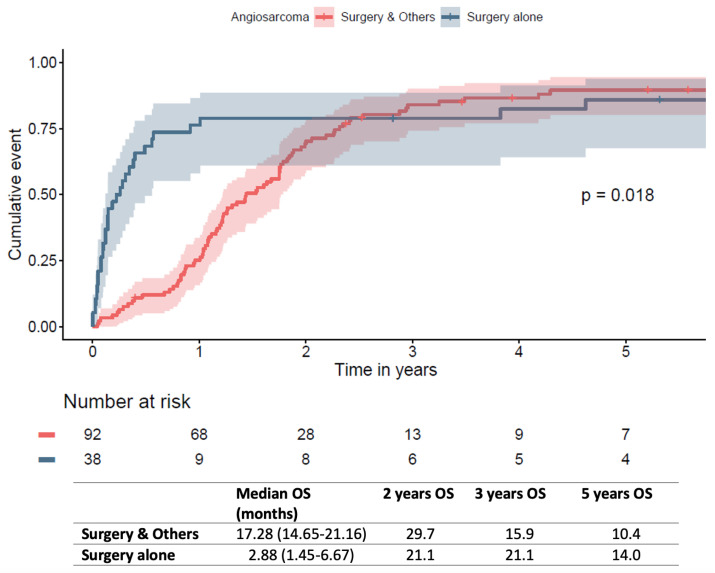
Kaplan–Meier curves for the death from any cause among surgery and others versus surgery-alone groups (landmark analysis revealed absence of statistical difference between both groups at ≥2.5 years cutoff (*p* = 0.41)).

The influence of other treatments over surgery on OS was further analyzed. The median OSs were 17.28 (95%CI: 14.65–21.16) and 2.88 (95%CI: 1.45–6.67) months for surgery and chemo-/radiotherapy and surgery-alone groups, respectively. The 2-, 3-, and 5-year OSs were 29.7%, 15.9%, and 10.4%, respectively, for surgery and chemo-/radiotherapy, while they were 21.1%, 21.1%, and 14.0% for surgery alone, respectively (log rank *p* = 0.018) (Figure 2).

With maximally selected rank analysis, 57 years of age resulted as the cutoff with the most significant relation to OS. Median OSs for age > 57 and age < 57 were 10.28 months (95%CI: 3.35–15.11) and 17.18 months (95%CI: 14.32–22.14), respectively. The 2-, 3-, and 5-year OSs were 17.78% vs. 32.2%, 13.33% vs. 20.1%, and 8.89% vs. 13.1% for age > 57 and age < 57, respectively (log-rank *p* = 0.012) (Figure S2).

**Figure 3:**
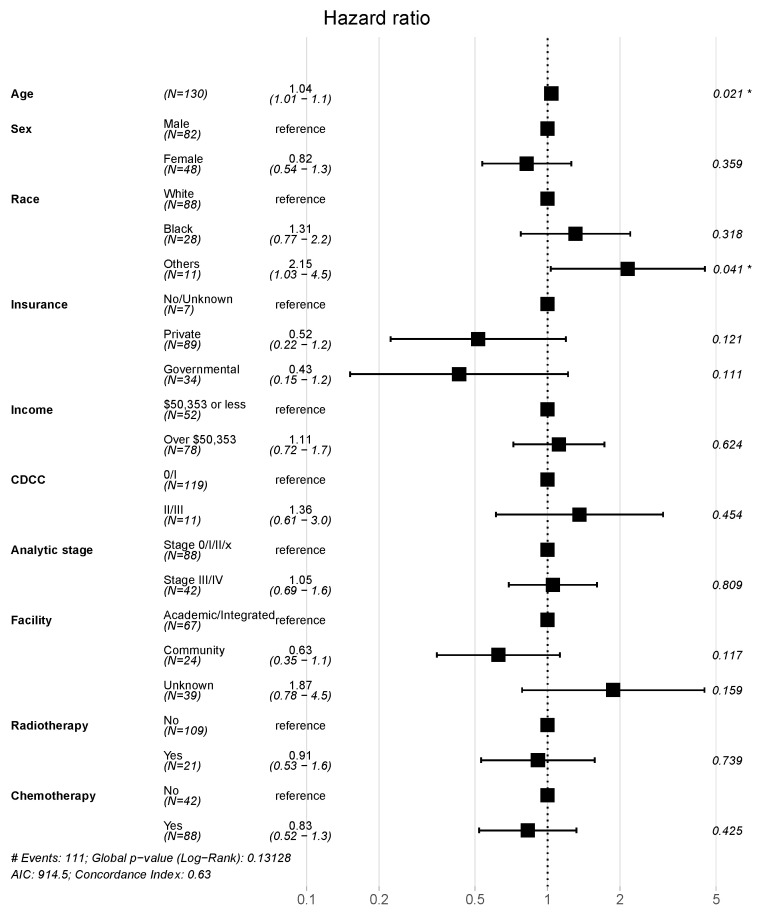
Forest plot of multivariate predictors of late mortality. # number of, * *p* < 0.05.

With multivariable Cox regression analysis, age (HR 1.04, 95%CI: 1.01–1.1, *p* = 0.021) and other race (HR 2.15, 95%CI: 1.03–4.5, *p* = 0.041) were identified as independent risk factors of late mortality (Figure 3).

## 4. Discussion

The main findings of our study showed that (1) overall, 30-day mortality was 10.8%, and the 2-, 3-, and 5-year OSs were 27.2, 17.4, and 11.4 months respectively; (2) multimodality treatment (surgery with chemo- and/or radiotherapy) reported longer survival rates when compared to surgery alone; (3) elderly patients presented the lowest survival rates, probably due to less combined therapy adoption.

Primary cardiac sarcomas are extremely rare tumors. As such, there is a paucity of information in the literature on these neoplasms, consisting only of observational studies/reports. Given this, clinical and prognostic information is scarce and prone to bias. While angiosarcoma is the most prevalent histology among cardiac sarcomas, in non-cardiac sarcomas, it is rarely encountered [9]. As is the case with other cardiac sarcomas, with angiosarcomas, tissue infiltration is common and metastatic spread is rapid, rendering surgical resection challenging [10]. Cardiac angiosarcomas’ expected mean survival without surgical resection has been reported to be 3.8 ± 2.5 months [13].

Surgical resection is considered to be the standard of care of treatment for primary cardiac sarcomas without metastasis at diagnosis [14]. Unfortunately, given the extremely invasive behavior of sarcomas, surgical resection is not always feasible. Indeed, in the case of cardiac angiosarcoma, a recent report has shown that the majority of cardiac angiosarcomas are managed by chemotherapy (65.0%), with patients undergoing surgery only 44.2% of the time [15]. Nevertheless, when possible, complete resection has been associated with a significant survival advantage, allowing survival beyond the short term [8,15]. If surgery is not possible (e.g., not eligible, patient’s death before surgery, patient refusal, etc.), patient mortality can approach up to 90% within the first year, independent of any post-surgical treatment [3,16]. Simpson et al. have shown that patients receiving complete surgical resection survived approximately double the time of patients without complete resection [17].

We have found that surgical treatment is associated with an OS of 14.3 months. This is in line with previous reports of patients undergoing resection by Antonuzzo et al. [18] and Hendriksen et al. [19] with a median OS of 14 and 16 months, respectively. Our findings should further encourage the use of surgery in treating such an aggressive disease whenever possible.

Patients with an age > 57 experienced lower OS compared to those with an age < 57 (log-rank = 0.012). This survival difference is in line with a previous report that analyzed age-related differences in primary malignant cardiac tumors [6]. Early (*p* < 0.001) and late (log-rank *p* < 0.001) survival outcomes were negatively impacted by increasing age. This may be partially explained by the difference in treatment strategies among age groups: patients with increasing age, less surgery (*p* = 0.037), and less chemotherapy (*p* < 0.001) were chosen.

Currently, no prospective trial has evaluated the effect of the use of additional therapy over and above surgery (chemotherapy, radiotherapy, or both). Moreover, differences between cardiac and non-cardiac sarcomas do not allow extrapolation of information from more powerful non-cardiac sarcoma studies. Thus, the information supporting these treatments remains scant and their use is highly controversial, especially considering that sarcomas in general are known to be chemo- and radiotherapy resistant [9,20]. Nonetheless, adjuvant chemotherapy may still be recommended even in the case of clear surgical margins [21].

A recent meta-analysis reported an improvement in mortality at 1 year, but no significant differences at 2, 3, 4, or 5 years between surgery only and multimodality groups [8]. Chemotherapy was identified as a protective factor for long-term mortality, while radiotherapy was not [6]. The current analysis on primary cardiac angiosarcomas suggests that chemotherapy (*p* = 0.425) and radiotherapy (*p* = 0.739) are not significantly associated with late survival with Cox regression. This is to be contrasted with several studies reporting a suggested benefit of combination therapy for treating cardiac angiosarcoma [9,22,23].

While surgery leads to a better prognosis for primary cardiac angiosarcoma, achieving complete resection often remains a complex option. The benefits of multimodality treatment in the setting of cardiac angiosarcoma are currently unknown, and no standardized chemotherapy regimen has yet been established.

### Limitations

This series illustrates the outcomes on cardiac angiosarcoma undergoing surgery with a multi-institutional series from the National Cancer Database but has several inherent limitations. The retrospective nature of our work may have introduced important biases and confounders that cannot be avoided even after statistical adjustments. The NCDB is subject to human error in coding and data input due to the multiple registrars. Furthermore, we could not investigate any genetic information related to angiosarcoma, as no such data are present in the NCDB. While diagnoses were confirmed with histological examinations after resection or biopsy, diagnostic imaging modalities, clinical presentation, tumor location within the heart, and name of chemotherapeutic regimen were not available. While it is conceivable that younger patients are advantaged by more aggressive resection surgery and more frequently treated with adjuvant therapy, the details of surgical resection were not mentioned. Despite several studies reporting a promising prognostic value for cardiac transplantation surgery [7,16], no such cases were encountered in our cohort. Heart transplantation has been proposed as a salvage approach for complex cardiac tumors where traditional surgical excision is not feasible. A recent meta-analysis estimated the prevalence of heart transplantation as a therapeutic option for primary malignant cardiac tumors at 1.44%, and 2.45% for cardiac tumors overall [1]. However, the benefits and effectiveness of this strategy remain a topic of controversy.

## 5. Conclusions

Primary cardiac angiosarcoma is a rare entity with late clinical manifestations and rapidly progressive nature that contribute to its unfavorable prognosis. Currently, no specific therapeutic approaches exist, and treatment typically adheres to guidelines established for sarcomas at other anatomical sites. Surgery remains the cornerstone of treatment, with adjuvant chemotherapy and/or radiotherapy considered for advanced local tumors. The findings of this study demonstrate that multimodality treatment is superior to surgery alone and is associated with improved survival outcomes. Notably, there were no discernible differences between short- and long-term survivor groups, with age emerging as a major predictor of enhanced survival in multivariate analysis. Further investigations are warranted to enhance our understanding of the behavior of primary cardiac angiosarcoma and to develop predictive models for long-term survival.

## Figures and Tables

**Table 1:** Criteria of the included patients with the entire group divided into short-term survivors (<2 years), intermediate survivors (2–3 years), and late-term survivors (≥3 years).

	Overall	Short Survivors	Intermediate Survivors	Late Survivors **	*p*	*p* (≥3 vs. <3 Years)
N	130	94	17	19		
Age (median [IQR])	50.5 [37.0, 62.0]	51.5 [37.0, 62.75]	48.0 [43.0, 58.0]	45.0 [35.5, 56.5]	0.510	0.264
Female sex	48 (36.9)	35 (73%)	6 (13%)	7 (15%)	0.999	0.999
Race (%)					0.487	0.375
· White	88 (69.3)	60 (68%)	14 (16%)	14 (16%)		
· Black	28 (22.0)	21 (75%)	2 (7.1%)	5 (18%)		
· Others	11 (8.7)	10 (91%)	1 (9.1%)	0 (0%)		
Insurance status					0.319	0.329
· No insurance/unknown	7 (5.4)	5 (71%)	2 (29%)	0 (0%)		
· Private	89 (68.5)	61 (69%)	12 (13%)	16 (18%)		
· Governmental	34 (26.2)	28 (82%)	3 (8.8%)	3 (8.8%)		
Median income quartile (%)					0.898	0.924
· Less than USD 40,227	16 (12.9)	11 (69%)	2 (13%)	3 (19%)		
· USD 40,227–USD 50,353	30 (24.2)	21 (70%)	5 (17%)	4 (13%)		
· USD 50,354–USD 63,332	34 (27.4)	26 (76%)	2 (5.9%)	6 (18%)		
· USD 63,333 or more	44 (35.5)	33 (75%)	5 (11%)	6 (14%)		
Urban/rural counties (%)					0.313	0.194
· Metropolitan	110 (87.3)	82 (75%)	13 (12%)	15 (14%)		
· Urban	11 (8.7)	9 (82%)	1 (9.1%)	1 (9.1%)		
· Rural	5 (4.0)	2 (40%)	1 (20%)	2 (40%)		
No high school graduate quartile (%)					0.923	0.675
· 17.6% or more	21 (16.9)	15 (71%)	3 (14%)	3 (14%)		
· 10.9%–17.5%	36 (29.0)	26 (72%)	4 (11%)	6 (17%)		
· 6.3%–10.8%	32 (25.8)	26 (81%)	3 (9.4%)	3 (9.4%)		
· Less than 6.3%	35 (28.2)	24 (69%)	4 (11%)	7 (20%)		
Great circle distance (miles; median [IQR])	18.30 [7.70, 50.80]	11.90 [6.50, 42.30]	38.50 [19.77, 629.75]	25.70 [5.65, 72.35]	0.057	0.874
CDCC (0 or 1/2 or 3) (%) *	119/11 (91.5/8.5)	85/9 (90.4/9.6)	15/2 (88.2/11.8)	19/0 (100/0)	0.357	0.366
Year of diagnosis (median [IQR])	2010 [2007, 2013]	2010 [2007, 2013]	2013 [2008, 2015]	2010 [2008.5, 2012]	0.112	0.560
Grade (poorly differentiated/anaplastic) (%)	63 (48.5)	46 (73%)	10 (16%)	7 (11%)	0.458	0.326
Tumor size (in mm; median [IQR])	60.0 [45.0, 85.0]	60.0 [48.5, 87.0]	48.0 [40.0, 74.75]	70.0 [50.25, 81.5]	0.437	0.606
Analytic stage group (%)					0.146	0.121
· Stage x/0/I	73 (56.2)	55 (75%)	11 (15%)	7 (9.6%)		
· Stage II	15 (11.5)	8 (53%)	3 (20%)	4 (27%)		
· Stage III/IV	42 (32.3)	31 (74%)	3 (7.1%)	8 (19%)		
Facility type (%)					0.173	0.797
· Academic/integrated	67 (51.5)	51 (76%)	7 (10%)	9 (13%)		
· Community	24 (18.5)	14 (58%)	7 (29%)	3 (13%)		
· Unknown	39 (30.0)	29 (74%)	3 (7.7%)	7 (18%)		
Radiation (%)	21 (16.2)	15 (71%)	1 (4.8%)	5 (24%)	0.251	0.192
Chemotherapy (%)	88 (67.7)	62 (70%)	15 (17%)	11 (13%)	0.107	0.426
30-day mortality (alive/dead) (%)	116/14 (89.2/10.8)	80/14 (85.1/14.9)	17/0 (100/0)	19/0 (100/0)	0.055	0.221
Vital status (alive/dead) (%)	16/114 (12.3/87.7)	1/93 (1.1/98.9)	4/13 (23.5/76.5)	11/8 (57.9/42.1)	<0.001	<0.001

This item provides a measure of the number of adults aged 25 or older in the patient’s zip code who did not graduate from high school and is categorized as equally proportioned quartiles among all US zip codes. * CDCC: Charlson/Deyo comorbidity condition. ** Percentage was calculated per row unless otherwise specified.

## Data Availability

We obtained approval to use the National Cancer Database, but it is not publicly available.

## Supplementary Materials

The following supporting information can be downloaded at: https://www.mdpi.com/article/10.3390/jcm12247764/s1, Figure S1: CONSORT flowchart of our included cohort; Figure S2: Kaplan–Meier curves for the death from any cause for age > 57 and age < 57.
