# Supplementary material for: Surgically Resected Cardiac Angiosarcoma: Survival Analysis from the National Cancer Database
Source: J Clin Med. 2023 Dec 18;12(24):7764. doi: 10.3390/jcm12247764 (PMC10744152; doi:10.3390/jcm12247764)

Figure S1: CONSORT flowchart of our included cohort

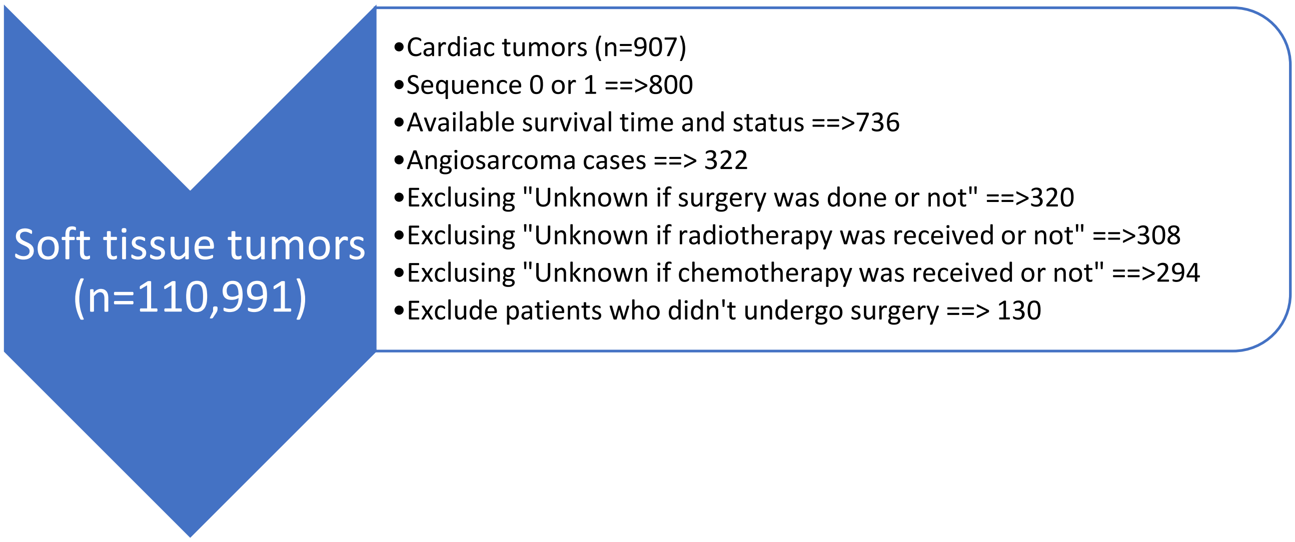

Figure S2: Kaplan Meier curves for the death from any cause for age > 57 and age <57

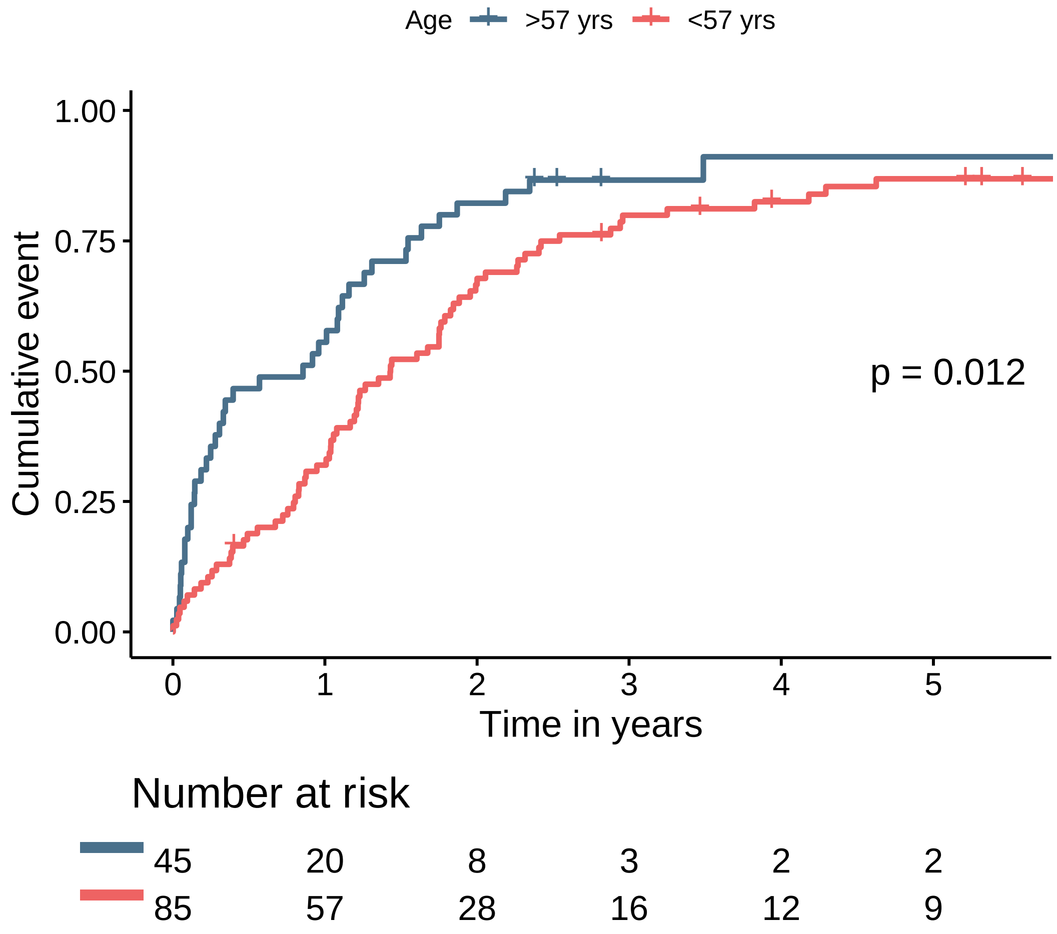

Supplement: Supplementary file 1 [file jcm-12-07764-s001.zip › jcm-2559721-supplementary.pdf]
